# SLEEP: A GEROSCIENCE TARGET

**DOI:** 10.1093/geroni/igae098.0582

**Published:** 2024-12-31

**Authors:** Judith Carroll

**Affiliations:** University of California, Los Angeles, Los Angeles, California, United States

## Abstract

Obtaining healthy quantity and quality of sleep is important in maintaining good mental and physical health, and cumulative evidence points to a role of sleep loss and sleep disturbances as a factor increasing age-related disease risk and progression. Many older adults experience less sleep on average per night than they obtained in earlier decades. Despite convincing epidemiologic data linking poor sleep and diseases of aging, the molecular pathways accounting for these links are not well delineated. Our work has begun to tackle these questions by examining how poor sleep may accelerate the biological aging process, while intervening to improve sleep may slow aging. We have shown that insomnia is related to biomarkers of aging including shortened telomere length, older epigenetic age, and a pro-inflammatory signal. Experimentally restricting sleep in older adults led to acute changes in gene expression indicating increased DNA damage response and senescence related inflammatory activation. Treating insomnia in older adults led to lower transcripts for cellular senescence and slowing of the pace of aging (DunedinPACE). Healthy sleep may provide biological advantage for increasing restoration and repair, while poor sleep may reduce the time the system can dedicate energy to repair of insults experienced during waking hours. Parallel to clearance of waste from the brain, we hypothesize that sleep is also important for physiological restoration throughout the body. Many unknowns still exist and future research will be needed to disentangle the mechanisms through which sleep alters biological aging.

